# Compartment Syndrome Secondary to *Bothrops* spp. Envenomation in Triângulo Mineiro, Region, Minas Gerais, Brazil

**DOI:** 10.1590/0037-8682-0130-2023

**Published:** 2023-07-24

**Authors:** Lucas Liporoni Toffano, Luiz Otávio da Silva, Fernando de Freitas Neves, Luciana de Almeida Silva Teixeira, Mario León Silva-Vergara

**Affiliations:** 1 Universidade Federal do Triângulo Mineiro, Departamento de Clínica Médica, Unidade de Doenças Infecciosas e Parasitárias, Uberaba, MG, Brasil. Universidade Federal do Triângulo Mineiro Departamento de Clínica Médica Unidade de Doenças Infecciosas e Parasitárias Uberaba MG Brasil

**Keywords:** Compartment Syndrome, Fasciotomy, Snake bite, *Bothrops* spp

## Abstract

**Background:**

Snakebite is a neglected global health problem with high morbidity. We describe compartment syndrome (CS) cases related to snakebites by *Bothrops* spp.

**Methods:**

The medical records of patients admitted with snakebites envenomation were reviewed.

**Results:**

Of 47 patients with *Bothrops* spp. envenomation (4 male; mean age: 42 years), 7 (15%) developed CS. The mean time to antivenom administration was 9.5 hours. The time to fasciotomy was variable. Seven patients developed infection and four had acute kidney injury.

**Conclusions:**

The incidence of CS is higher than that reported previously. This may be due to the clinical severity and long delay before administering antivenom.

Snakebite envenomation meets the World Health Organization (WHO) criteria for a neglected tropical disease. Globally, 5.4 million people are bitten by snakes annually, whereas 2.7 million have evidence of snakebites with envenomation. Consequently, each year, between 81,000 and 139,000 deaths are caused directly by snakebite envenomation, and the incidence of amputation and permanent sequelae due to snakebite is approximately three times this figure. Most of these cases are restricted to Africa, Asia, and Latin America^1^^,^^2^.

From 2000 to 2017, 471,801 cases of snakebite envenomation were reported in Brazil, with an annual average of 27,000 cases and 1,892 snakebite-related deaths during this period. Snakes belonging to the genera *Bothrops* (pit vipers) and *Crotalus* (rattlesnakes) represent 90% and 7% of the reported cases of envenomation, respectively. The incidence is highest in the Northern and Midwestern regions of the country (49.2 and 19.7 cases per 100.000 population, respectively)^3^.

The venom of snakes of the genus *Bothrops* contains a mixture of metalloproteases, serine proteases, collagenases, L-amino-acid oxidases, phospholipase A2, and hyaluronidases, which cause myonecrosis and dermatonecrosis. Toxic muscle lesions are mediated by metalloproteases, phospholipase A2, and serine proteases, and the direct effect of venom at the capillary level. Experimental models have shown that damage to the vascular endothelium causes extravasation of plasma and leads to inflammation, edema, and ecchymosis^4^^,^^5^^,^^6^.

The severity and outcome of snakebite envenomation are related to the aggressor species, its size and effectiveness at inoculating venom, age of the victim, size of the affected limb, elapsed time between the bite and administration of specific antivenom, and quantity of venom inoculated^7^^,^^8^.

*Bothrops* spp. envenomation leads to myonecrosis and secondary infection, acute kidney injury, and, less commonly, shock and compartment syndrome (CS).The late is rare and most data in the literature are from case reports or small case series. Two recent publications described 17 (10.8%) and 9 (9.7%) cases of CS among 158 and 92 patients, respectively^8^.

According to several studies, CS manifests as intense, disproportionate, and progressive pain that is refractory to common analgesics, with extensive edema, paresthesia, poikilothermia, pulselessness, paralysis and cutaneous pallor^8^^,^^9^^,^^10^.

Of these signs and symptoms, severe pain is the most reliable early indicator of CS given that the signs of ischemia occur much later^,^^7^^,^^8^^,^^11^. The genesis of pain through nociceptive transmission from the peripheral to central nervous system is mediated by biogenic amines, nitric oxide, and eicosanoids, and nociceptor sensitization by nitric oxide and eicosanoids. Phospholipase A2 also plays a crucial role^4^^,^^5^^,^^11^.

This study aimed to present the epidemiological, clinical, and outcome data of a case series of patients with CS associated with *Bothrops* spp. envenomation over one year in a reference Teaching Hospital in Minas Gerais, Brazil.

From January 30, 2018, to January 30, 2019, patients with snakebite envenomation admitted to the emergency department of the Teaching Hospital of the Federal University of Triângulo Mineiro in Uberaba-MG were prospectively evaluated. Individuals of both sexes aged greater than 18 years who agreed to participate in the study were included. Clinical, epidemiological, and outcome data were obtained from clinical interviews and medical record review.

The patients were clinically evaluated and followed up daily by the same medical team until hospital discharge. The characterization of the type of envenomation was based on the signs and symptoms presented by the patients, and the laboratory data. The clinical classification and specific antivenom therapy were based on the guidelines of the Brazilian Ministry of Health for venomous animals. The snake species was not identified, nor was the antigenemia test performed. In patients who presented with clinical worsening or persistence of abnormal coagulation parameters after 12-24 hours, additional vials of specific antivenom were administered. The case definition of CS was based on evidence of two or more clinical criteria, such as extensive and hardened edema sensitive to digital pressure; intense, disproportionate, progressive pain refractory to common analgesics; paresthesia; poikilothermia; pallor; and pulselessness of the affected limb^7^^,^^8^.

The clinical, epidemiological, and outcome data of the patients were analyzed using descriptive statistics. The study was approved by the institutional ethical review board (protocol number 248340).

During the one-year study period, 54 patients with snakebite envenomations were admitted to the emergency department. Of these, 47 (87.0%) were bitten by *Bothrops* spp. and 7 (13.0%) by *Crotalus* spp. Forty-two patients (77.7%) were male, and patients had a median age of 45 years. The elapsed time between the snakebite and administration of emergency care was up to 3 hours in 29 cases (53.3 %). The legs were affected in 38 (70.3%) patients. Twenty-two (40.7%) and 14 (26%) patients were classified as having moderate and severe envenomation, respectively. 

The mean time between the snakebite and administration of specific antivenom was 6 hours. The mean number of antivenom vials administered was eight. Coagulation abnormalities were observed in 36 patients (67%). Seventeen (31%) patients required additional antivenom vials (half of the previous dose) because of clinical worsening. The mean length of hospital stay was 6 days.

Of the 47 (87.0%) patients who presented with envenomation by snakes of the *Bothrops* genus, 20 (42.5%), 12 (25.5%), and 7 (15%) presented with secondary infection, acute kidney injury, and CS, respectively. Patients with evidence of local infection received ceftriaxone and clindamycin for 7 days. Of the 12 patients with acute kidney injury, three required hemodialysis.

Of the seven patients with CS, the majority were male, with a mean age of 42 years. The upper and lower limbs were affected in four and three cases, respectively. Clinically, four cases were considered moderate envenomation and three were considered severe envenomation. The seven patients presented with extensive and hardened edema, cutaneous hyperesthesia, and severe disproportionate pain (Table 1).

**TABLE 1:** Epidemiological, clinical and outcome data of seven patients with compartment syndrome associated to *Bothrops* spp. envenomation.

Case	Age (years)	Sex	Elapsed time between snakebite and antivenom administration (hours)	Time from admission to fasciotomy (hours)	Complications	Outcome
**1**	67	M	10	4	BI +AKI*	Good
**2**	54	F	20	12	BI + AKI*	Good
**3**	40	F	5	15	BI	Good
**4**	46	M	10	1	BI +	Good
**5**	46	M	7	07	BI + AKI	Good
**6**	18	M	9	19	BI + AKI	Good
**7**	22	M	6	48	BI	Good

**BI:** Bacterial infection; **AKI:** Acute kidney injury; **M:** male; **F:** female. * Hemodialysis.

The mean time between snakebite and antivenom administration was 9.5 hours, and the time from admission to fasciotomy ranged from 1 to 48 hours (Figure 1). All seven patients presented with secondary infection, and four had acute kidney injury. The mean length of hospital stay was 3 weeks. All patients had good outcomes without apparent sequelae (Table 1). According to the WHO, snakebite envenomation is a neglected disease; most cases occur in Latin America, Asia, and Africa, where several genera and species of snakes are endemic, resources are limited, and medical and social costs secondary to local and systemic complications derived from envenomation are underestimated^1^^,^^2^. Snakes of the genera *Bothrops* and *Crotalus* account for most cases of snakebite in Latin America^3^.

**FIGURE 1:**
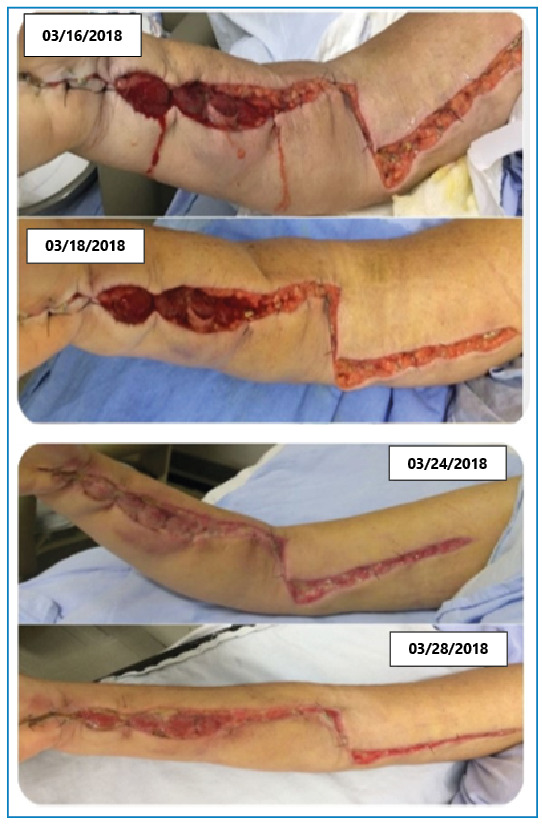
Sequencial photos of a woman aged 54 years with severe compartment syndrome caused by a bite by *Bothrops* spp.

This report describes seven cases of CS among 47 patients who presented clinical and laboratorial evidence of envenomation by *Bothrops* spp. and were admitted to a teaching hospital in the Triângulo Mineiro region during a one-year period. Previous epidemiological data showed that *Bothrops moojeni, Bothrops neuwiedi* and *Bothrops alternatus* are endemic in the region ^12^^,^^13^. Over the past three decades, local notification records have shown an average of 80 snakebite cases per year, and the occurrence of CS has been sporadic.

The figure of 15% CS observed in this study is higher than that previously reported from Brazil and elsewhere. Previous reports have described CS due to envenomation by several genera and species of snake, and the time periods of observation were very variable (Table 2). Most patients described in this report were admitted and treated at night or during weekends and underwent fasciotomy before additional doses of antivenom were administered, as recommended by some authors^15^. However, on admission, these patients presented with signs and symptoms of moderate or severe envenomation, and there was a long delay between the snakebite and administration of the antivenom (Table 1).

**TABLE 2:** Update of some case-series of compartment syndrome associated with snakebite envenomation.

Number of cases of snakebite envenomation	Observation period (years)	Cases of compartment syndrome N (%)	Snake genus and species	Reference
92	3	9 (9.7%)	*Bothrops* spp.	9
			*Lachesis* spp.	
			*Elapidic* spp.	
307	6	4 (1.3%)	*Bothrops* spp.	10
			*Lachesis* spp.	
158	6	17 (10.7%)	*Gloydius* spp.	8
			*Rhabdophis tigrinus*	
73*	16	3 (4.1%)	*Bothrops jararaca*	14
			*Bothrops alternatus*	
			*Bothrops neuwiedi*	
136	4	9 (6.6%)	*Trimeresurus* spp.	7
			*Daboia russelli formosensis*	
			*Naja naja atra*	
			*Bungarus multicinctus*	
			*Deinagkistrodon acutus*	
47	1	7 (14.8%)	*Bothrops* spp.	Present report

* Pediatric Cases.

Although snake identification was not performed, the severity of CS suggests *B moojeni* as the main associated species because it has been reported as the predominant species in the region^12^^,^^13^. The venom of this species is considered more potent than that of *B alternatus,* and the presence of metalloproteases and phospholipase A2 corresponds to the severity and extent of tissue damage. Other venom components include serine proteases associated with local lesions^4^^,^^5^^,^^6^.

The severity of the envenomation can also be associated with the time elapsed between the snakebite and the administration of antivenom, which was 9.5 hours for the seven patients with CS compared with an average of 6 hours for the remaining patients admitted with snakebite envenomation. Although acute kidney injury and secondary infection occurred in most patients with CS, all patients had good outcomes without permanent sequelae.

In a recently published case series of 158 patients seen over 6 years, 33 were clinically suspected to have CS caused by other genera and snake species. Of these cases, 17 (10.8%) had CS confirmed and underwent fasciotomy based on the criteria of increased intracompartmental pressure (IP) measured using the Whiteness method, and lack of response to the additional doses of antivenom^8^. The high incidence of CS in this study was attributed to the long delay in administration of antivenom and to the involvement of the arm in most cases, where the venom can be inoculated more easily and deeper into the muscle compartment.

Compartmental syndrome associated with snakebite occurs due to the toxic effect of the venom on tissues, which leads to edema and inflammation within the muscle compartment surrounded by untensible tissues, such as ligaments, bone, and muscle fascia. Furthermore, the progression of edema leads to increased IP and reduced tissue perfusion, local ischemia, necrosis, and neurovascular compromise. Thus, a vicious cycle is created in which compression leads to hypoxia and tissue acidosis, which increase capillary permeability, inducing worsening edema^6^^,^^10^^,^^11^.

Several animal studies have suggested that elevation of IP occurs after intramuscular inoculation of the venom with the release of tissue fluids into the compartment, which represents severe poisoning, but this is not the cause of the complication. If venom plays a major role in this event, then the treatment is the administration of specific antivenom^15^.

A review of 99 publications on the effectiveness of fasciotomy for treating CS in animals and humans was reported and pointed out eight controlled experimental studies in animals that showed that antivenom reduces IP and increases tissue perfusion, whereas fasciotomy either did not benefit or worsen myonecrosis. The authors concluded that even in exceptional cases of confirmed CS, the treatment should be to repeat the dose of antivenom and postpone fasciotomy until evidence is obtained that the patient does not improve with these measures^16^.

The cases of CS described in this report were diagnosed based on clinical criteria, and IP was not measured because a specific device for measuring IP was unavailable at the time. The epidemiological, clinical, and outcome features of these patients are in accordance with other reports, although, to our knowledge, the incidence of CS of 15% is the highest reported to date, and is likely to be attributable to the severity of the envenomation, and the time elapsed between the snakebite and antivenom administration. However, there may have been selection bias during the clinical evaluation because it is difficult to distinguish a severe case of snakebite from snakebite with CS based on clinical criteria alone. According to several authors, careful observation of patients with snakebite envenomation admitted to the emergency department during the first hours after initial management, and repetition of antivenom administration when clinical worsening occurs are crucial and can prevent or decrease the need for fasciotomy in most cases^8^^,^^14^.
